# ﻿Afrotropical *Centistidea* Rohwer, 1914 (Hymenoptera, Braconidae) with description of four new species

**DOI:** 10.3897/zookeys.1216.133127

**Published:** 2024-10-22

**Authors:** Zhen Liu, Andrew Polaszek

**Affiliations:** 1 Changde Key Innovation Team for Wetland Biology and Environmental Ecology, College of Life and Environmental Sciences, Hunan University of Arts and Science, Changde 415000, China; 2 Natural History Museum, London, UK; 3 State Key Laboratory of Development Biology of Freshwater Fish Sub-Center for health aquaculture, Hunan University of Arts and Science, Changde 415000, China

**Keywords:** Africa, leaf-miner, Miracinae, *Mirax*, new taxa

## Abstract

The braconid parasitoid wasp genus *Centistidea* Rohwer, 1914 is revised for the Afrotropical region, with four new species described; *Centistideaareolaris* Liu & Polaszek, **sp. nov.**, *C.linearis* Liu & Polaszek, **sp. nov.**, *C.longipedes* Liu & Polaszek, **sp. nov.**, and *C.turneri* Liu & Polaszek, **sp. nov.** are described based on specimens from the Natural History Museum, United Kingdom, and the Royal Museum for Central Africa, Belgium. An illustrated key to species in the Afrotropical region is provided.

## ﻿Introduction

Miracinae (Hymenoptera, Braconidae) are quite rare in all collections worldwide, with only 70 species known from all geographical regions (Yu et al. 2016; Ghramh et al. 2019; Ranjith et al. 2019, 2023; Slater-Baker et al. 2022). Three miracine genera have been described: *Mirax* Haliday, 1833, *Centistidea* Rohwer, 1914, and *Rugosimirax* Ranjith & van Achterberg, 2023 (Yu et al. 2016; Ranjith et al. 2023; Liu and Polaszek 2024). The miracine fauna of the Afrotropical region is only by the report of four species in the genus *Centistidea*, viz., *C.africana* (Brues, 1926), *C.leucopterae* (Wilkinson, 1936), *C.mubilibana* (de Saeger, 1944), and *C.tihamica* Ahmad & Pandey, 2019. The first three species listed above were originally placed in *Mirax*, but this generic placement was questioned recently (Liu and Polaszek 2024).

Here we describe four new species of *Centistidea* from Cameroon, South Africa, and Uganda, together with a preliminary revision of this group in the Afrotropical region as part of our ongoing project on worldwide Miracinae.

## ﻿Materials and methods

Specimens studied are deposited in the Natural History Museum, UK (**NHMUK**) and the Royal Museum for Central Africa, Belgium (**RMCA**). Descriptions and measurements were made using a stereomicroscope (Zeiss® Stemi SV6). Photographs were taken and processed using a digital camera (Zeiss AxioZoom combined with Helicon software or Hirox HRX-01). The images were further processed using Adobe Photoshop® CS6. Morphological terms for body structures and measurements mainly follow Ranjith et al. (2023) and Slater-Baker et al. (2022). The wing vein terminology follows the modified Comstock-Needham system (van Achterberg 1993). The terminology of the cuticular sculpture follows Harris (1979). Abbreviations used in this research are as follows: POL = postocellar line, OOL = ocular-ocellar line, OD = ocellar diameter; T1 = 1^st^ tergite of metasoma, T2 = 2^nd^ tergite of metasoma, T3 = 3^rd^ tergite of metasoma.

## ﻿Taxonomy

### ﻿Key to species of *Centistidea* from the Afrotropical region

**Table:** 

1	Propodeum with lateral carinae alongside median longitudinal carina, and with reticulate sculpture between lateral carinae (Fig. 1k)	**2**
–	Propodeum without lateral carinae alongside median longitudinal carina, at most with indistinct punctures anterolaterally (e.g. Figs 2h, 3i, 4j, 6h)	**3**
2	Scutellar sulcus greatly reduced and not impressed; temple obliquely narrowed behind eyes; vein 1-CU1 as long as 2-CU1	***C.africana* (Brues, 1926)**
–	Scutellar sulcus obviously depressed (Fig. 1e); temple roundly narrowed behind eyes (Fig. 1b); vein 1-CU1 0.6 × length of 2-CU1 (Fig. 1g)	***C.areolaris* Liu & Polaszek, sp. nov.**
3	Scutellar sulcus not depressed (Fig. 2e); temple strongly constricted behind eyes (Fig. 2b); eyes nearly 4.0 × longer than temple in dorsal view (Fig. 2b)	***C.leucopterae* (Wilkinson, 1936)**
–	Scutellar sulcus obviously depressed, even when with crenualation (e.g. Fig. 3e); temple less constricted behind eyes (e.g. Fig. 3b); eyes 1.5–2.0 × longer than temple in dorsal view (e.g. Fig. 3b)	**4**
4	Propodeum rugulose with median carina bifurcated at apical third (Fig. 5d); vein r of fore wing hardly visible (Fig. 5e)	***C.mubilibana* (de Saeger, 1944)**
–	Propodeum smooth with median carina bifurcated medially (e.g. Fig. 6h) or nearly to apex with some transverse rugae beside the median carina (e.g. Figs 3i, 4j); vein r of fore wing developed (e.g. Figs 3g, 4g, 6g)	**5**
5	Medio-posterior depressions of scutellum distinctly separated (Fig. 3e)	**6**
–	Medio-posterior depressions of scutellum touching each other (Figs 4e, 6e)	**7**
6	Vein 1-SR of fore wing absent; vein r of fore wing very prominent; median longitudinal carina bifurcated at middle of propodeum	***C.tihamica* Ahmad & Pandey, 2019**
–	Vein 1-SR of fore wing present (Fig. 3g); vein r of fore wing less prominent (Fig. 3g); median longitudinal carina bifurcated at nearly apical extremity of propodeum (Fig. 3i)	***C.linearis* Liu & Polaszek, sp. nov.**
7	Propodeum with regular short transverse rugae along median carina (Fig. 4j); T3 polished (Fig. 4k); vein 1-R1 of fore wing present (Fig. 4g)	***C.longipedes* Liu & Polaszek, sp. nov.**
–	Propodeum without regular short rugae along median carina (Fig. 6h); T3 longitudinally striate (Fig. 6k); vein 1-R1 of fore wing absent (Fig. 6g)	***C.turneri* Liu & Polaszek, sp. nov.**

#### ﻿ Centistidea africana

Taxon classificationAnimaliaHymenopteraBraconidae

(Brues, 1926)

9E3D6039-00B9-59DF-8BA4-11E98CEDC650

Mirax africana Brues, 1926: 292. Holotype in Durban Museum and Art Gallery, Durban, South Africa (not examined).
Mirax africana : De Saeger 1944: 37; Shenefelt 1973: 676.

##### Diagnosis.

Body length 1.7 mm, yellow-brown; occiput deeply emarginate; head matte, without median groove on vertex; ocelli in small equilateral triangle, about the distance to each eye; antenna shorter than body, first three flagellomeres of equal length, the fourth and following becoming shorter and more slender; notauli very distinct anteriorly, less so behind; mesoscutum and central part of scutellum minutely granular, matte; scutellar sulcus greatly reduced and not impressed; scutellum depressed at sides, with a large subtriangular, smooth, margined impression on each side, and a pair of small round foveae at apex, the two enclosed together in an oval margined line; propodeum with distinct median and a lateral longitudinal carina, more or less irregularly reticulate between the carinae, more coarsely posteriorly; T1 narrow; pterostigma less than half as wide as long, with vein r emitted from its middle, 1-CU1 as long as 2-CU1 (following Brues 1926).

##### Distribution.

South Africa.

##### Host.

Unknown.

##### Note.

No specimens were available for this study.

#### ﻿ Centistidea areolaris

Taxon classificationAnimaliaHymenopteraBraconidae

Liu & Polaszek sp. nov.

BC84D8AC-9D64-5E0C-9686-B868BA97E471

https://zoobank.org/712D4603-E764-40FD-86C9-821AD4505472

Fig. 1

##### Diagnosis.

Body length 2.0 mm, light red-brown; eyes 1.8 × longer than temple in dorsal view; temple smooth, superficially punctate, indistinctly constricted behind eyes in dorsal view; hind ocelli in a shallow depression, distance between fore and a hind ocellus 1.2 × longer than minor axis of a hind ocellus, POL:OD:OOL = 1.3:1.0:2.9; vertex between eye and hind ocellus nearly smooth except some extremely fine transverse wrinkles; face nearly polished except some punctures along eyes, not convex medially, 1.5 × wider than high; antenna nearly as long as body length, with 1^st^, 2^nd^, penultimate and ultimate flagellomeres 4.9, 5.5, 2.7 and 3.0 × longer than wide, 1^st^ indistinctly longer than 2^nd^; mesoscutum with superficial and weakly-defined punctures anteriorly and laterally, largely smooth dorsally, notauli less obvious, weakly crenulated near to anterior 1/3; scutellar sulcus concave but not crenulated; medio-posterior depressions of scutellum large and oblong, both enclosed by a margined line; propodeum with distinct median carina and carinate-areolate elements medio-apically; pterostigma narrow, 2.9 × as long as its widest part; vein 1-R1 attenuated to 0.3 of length of pterostigma; T1 3.9 × longer than its maximum width, radially striate at lateral membranous area; T2 triangular part 1.3 × wider than median length; T3 1.9 × longer than T2.

##### Description.

**Figure 1.**
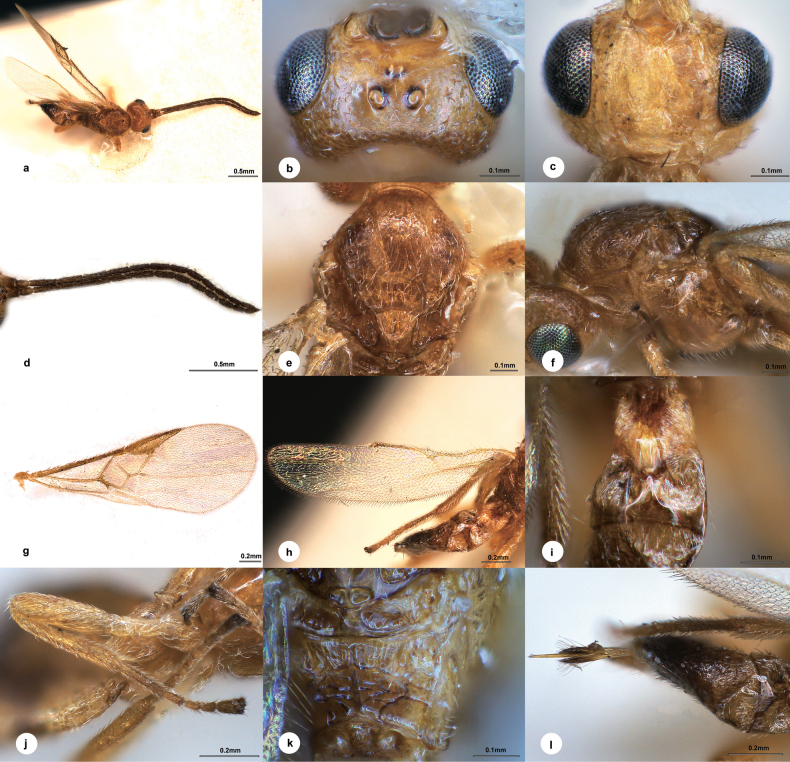
*Centistideaareolaris* Liu & Polaszek, sp. nov., female, holotype **a** habitus, dorsal view **b** head, dorsal view **c** head, frontal view **d** antenna **e** mesosoma, dorsal view **f** mesosoma, lateral view **g** fore wing **h** hind wing **i** T1–T3, dorsal view **j** hind leg **k** propodeum, dorsal view **l** ovipositor sheath.

**Female.** Body length 2.0 mm, fore wing length 2.5 mm (Fig. 1a).

***Head*.** 1.8 × as wide as long, 1.2 × wider than mesoscutum. Eyes 1.8 × longer than temple in dorsal view (Fig. 1b). Temple a little shiny, smooth, superficially punctate, sparsely pubescent, indistinctly constricted behind eyes in dorsal view. Ocelli small, hind ocelli in a shallow dimple, distance between fore and a hind ocellus 1.2 × longer than minor axis of a hind ocellus, POL:OD:OOL = 1.3:1.0:2.9. Frons flat and polished. Vertex between eye and hind ocellus shiny and nearly smooth except some extremely fine transverse wrinkles. Face (Fig. 1c) shiny, nearly polished except some punctures along eyes, not convex medially, transverse, 1.5 × wider than high. Clypeus 2.0 × wider than medial length, nearly polished. Length of malar space 1.4 × longer than width of mandible. Antenna (Fig. 1d) nearly as long as body length, with scape, pedicel and 1^st^, 2^nd^, penultimate and ultimate flagellomeres 1.4, 1.6, 4.9, 5.5, 2.7 and 3.0 × longer than wide, 1^st^ indistinctly longer (nearly 1.1 ×) than 2^nd^, flagellomeres gradually shortened to apex.

***Mesosoma*.** Length:width:height = 1.4:1.0:1.2. Mesoscutum (Fig. 1c) shiny with superficial and weakly defined punctures anteriorly and laterally, largely smooth dorsally, notauli less obvious, weakly crenulated near to anterior 1/3. Scutellar sulcus slightly curved, concave, not crenulated. Scutellum shiny, sculptured as dorsal mesoscutum, medio-posterior depressions large and oblong, both enclosed by a margined line. Propodeum (Fig. 1k) shiny with distinct median carinae reaching posterior margin, rugulose anteriorly, with carinate-areolate elements medio-apically. Mesopleuron (Fig. 1f) highly polished, impunctate.

***Legs*.** Hind femur (Fig. 1j) 3.7 × as long as its widest part. Length of hind femur:tibia:basitarsus = 2.0:2.4:1.0. Basitarsus of hind leg 0.6 × as long as tarsomeres 2–5.

***Wings*.** Fore wing (Fig. 1g): pterostigma narrow, 2.9 × as long as its widest part (Fig. 1b); vein 1-R1 attenuated to 0.3 length of pterostigma; vein r:2-SR:2-M = 1.0:7.7:3.0, 1-SR:1-M = 1.0:6.1, 1-CU1:2-CU1 = 1.0:1.7; first discal cell of fore wing nearly 1.2 × wider than high. Hind wing (Fig. 1h): vein M+CU:1-M:r-m = 2.0:2.2:1.0.

***Metasoma*.** 0.9 × length of mesosoma. T1 (Fig. 1i) highly polished, spatula-shaped, 3.9 × longer than its maximum width, distinctly narrowed anterior-medially, radially striate at lateral membranous area. T2 triangular part 1.3 × wider than median length, longitudinally striate at lateral membranous area. T3 1.9 × longer than T2, weakly longitudinally striate. Hypopygium shorter than length of metasoma. Ovipositor sheath (Fig. 1l) 1.3 × longer than hind basitarsus, with long and dense setae apically.

***Colour*.** Light red-brown, except apex of metasoma darker brown (Fig. 1a). Palpi and spurs pale yellow. Antenna and apical ovipositor sheath dark brown. Legs yellow except apical tarsomeres. Wing membrane hyaline, pterostigma yellow-brown, vein 1-SR, 1-M and 1-CU1 brown, other veins brown.

**Male.** Unknown.

##### Host.

Unknown.

##### Material examined

**(NHMUK). *Holotype***: • 1♀, South Africa, Port St. John, Pondoland, RE Turner, 12–30.VI.1923, Brit. Mus 1923-363, No. NHMUK010639675. ***Paratype***: • 1♀, same data except IX.1923, Brit. Mus 1923-510, No. NHMUK010639676.

##### Distribution.

South Africa.

##### Etymology.

The specific name “*areolaris*” refers to the propodeum with carinate-areolate elements medio-apically.

##### Remarks.

This species is similar to the Neotropical species, *C.vertus* (Papp, 2013). Its peculiar propodeum is very rare in Miracinae with both median carina and areola present, but differs in the following: antenna slightly shorter than body length, with penultimate flagellomere 2.7 × longer than wide (antenna 1.2 × longer than body length, with penultimate flagellomere 4.0 × longer than wide in *C.vertus*); medio-posterior depressions on scutellum distinct in an enclosed oval margined line (absent in *C.vertus*); and T2 gradually wider basally (narrowly parallel-sided basally in *C.vertus*).

#### ﻿ Centistidea leucopterae

Taxon classificationAnimaliaHymenopteraBraconidae

(Wilkinson, 1936)

E12DEC9F-0E40-5F4A-8CF2-537A146490DF

Fig. 2

Mirax leucopterae Wilkinson, 1936: 385. Holotype in NHMUK, examined.
Mirax leucopterae : De Saeger 1944: 36; Decelle 1962: 189; Nixon 1965: 9; Shenefelt 1973: 678.

##### Diagnosis.

**Figure 2.**
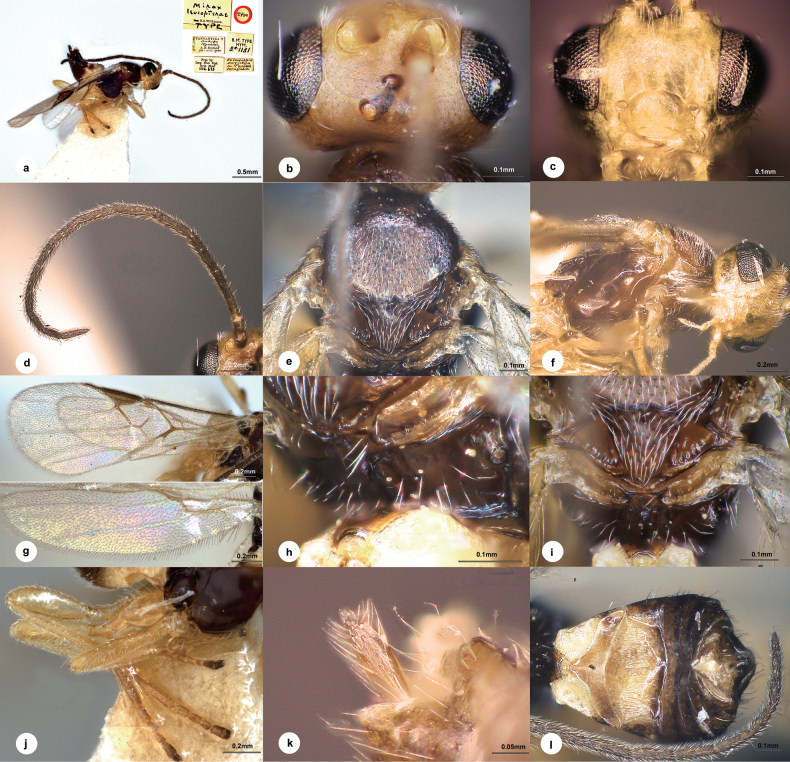
*Centistidealeucopterae* (Wilkinson, 1936), female, holotype **a** habitus, lateral view **b** head dorsal view **c** head, frontal view **d** antenna **e** mesosoma, dorsal view **f** mesosoma, lateral view **g** wings **h** propodeum **i** scutellum **j** hind leg **k** ovipositor sheath **l** metasoma, dorsal view.

Body length 1.5–2.0 mm, mostly black (Fig. 2a); head exceedingly minutely punctate, more densely so on the face than on the frons and vertex (Fig. 2b, c); ocelli in a small equilateral triangle which about the length to each eye; flagellum of female rather shorter, and of male rather longer, than body; mesoscutum and disc of scutellum (Fig. 2e) throughout regularly, minutely punctate; notauli very distinct on anterior declivity; scutellar sulcus greatly reduced; propodeum (Fig. 2h) with a very strong median longitudinal carina, which bifurcates at about the middle and forms a pair of very strong, diverging carinae, punctate and setiferous anterolaterally, impunctate elsewhere; vein r (Fig. 2g) emitted before middle of pterostigma; T1 long and narrow, broadened in apical half; T2 (Fig. 2l) entirely smooth except two or three minute punctures, membranous lateral basal areas with fine longitudinal aciculation; T3 with some minute punctures across apical third or fourth, otherwise almost throughout with fine longitudinal aciculation.

##### Material examined.

***Holotype***: • 1♀, [Tanzania] Tanganyika T[erritory], Bukoba, Nyakato, AH Ritchie, 24.VIII.1935, ex *Leucopteradaricella* on *Pavettaternifolia*, type No. B.M.TYPE HYM. 3.C.1131, No. NHMUK010639681; ***paratypes***: • 9♂♂, same data as holotype Nos. NHMUK010635732, 010639360, 010639353, 010639367, 010639480, 010639377, 010639403, 010639362, 010639384.

##### Other materials.

• 3♀♀3♂♂, Madagascar, Fianarantsoa, 19.III.1968, C.I.E. A2287, ex *Leucoptera*, det. Nixon, 1968, Nos. NHMUK010639677 (2), 010639678 (2), 010639679 (2); • 3♀♀, Madagascar, Tulear Berenty 12 km, N.W. Amboasary, JS Noyes, MC Day, 5-15.V.1983, B.M.1983-201, Nos. NHMUK010639735, 010639730, 010639744; • 1♀, Kenya, Diani Beach, VII.1951, NLH Krauss, B.M.1951-541, No. NHMUK010639745; • 1♀, South Africa, Port St. John, Pondoland, 25-31.III.1923, RE Tuner, Brit.Mus. 1923-241, No. NHMUK010639726; • 1♀3♂♂, Zimbabwe Chipinga Dist., Masasimn, 20.VII.1990, ex larvae of *Leucopterameyricki*, IIE 21643, det. AK Walker, 1991, No. NHMUK010639680.

##### Host.

*Crobylophoradaricella* [*Pavettaternifolia*], *Leucoptera* sp. [*Cremaspora*, *Cremasporahirsutus*, *pavetta*], *Leucopteracoffeella*, and *Leucopteracoma* (Yu et al. 2016; Decelle 1962).

##### Distribution.

New records for Kenya, Madagascar, South Africa, Zimbabwe; Democratic Republic of Congo, Tanzania.

#### ﻿ Centistidea linearis

Taxon classificationAnimaliaHymenopteraBraconidae

Liu & Polaszek sp. nov.

35CDA258-4BDF-5A3B-A82C-084086F4E380

https://zoobank.org/AB062463-62D2-4E1B-9483-8F2076BD8851

Fig. 3

##### Diagnosis.

Body length 2.6 mm, dark brown; head 1.7 × as wide as long, 1.5 × wider than mesoscutum; eyes 2.0 × longer than temple in dorsal view; temple slightly shiny, small setose punctures with transverse wrinkles in between, not constricted behind eyes in dorsal view; distance between fore and a hind ocellus 1.5 × longer than minor axis of a hind ocellus, POL:OD:OOL = 1.3:1.0:3.1; clypeus 1.6 × wider than medial length, weakly defined punctate; antenna 1.2 × longer than body length, with 1^st^, 2^nd^, penultimate and ultimate flagellomeres 5.2, 4.9, 4.1 and 4.3 × longer than wide, 1^st^ about as long as 2^nd^; mesoscutum with superficial and extremely small punctures, intervals with extremely fine wrinkles, notauli obvious, crenulated near to anterior 1/2; scutellar sulcus slightly curved, indistinctly crenulated; medio-posterior depressions of scutellum large and oblong, interval 3/4 of depression diameter; propodeum with distinct median carinae reaching beyond weak defined costulae, anterior parts with indistinct punctures anteriorly except wrinkles elsewhere as posterior parts, anterior part 2.8 × longer than median length of metanotum; vein 1-R1 0.3 of length of pterostigma; T1 2.8 × longer than its maximum width; T2 2.6 × wider than median length; T3 1.7 × longer than T2.

##### Description.

**Figure 3.**
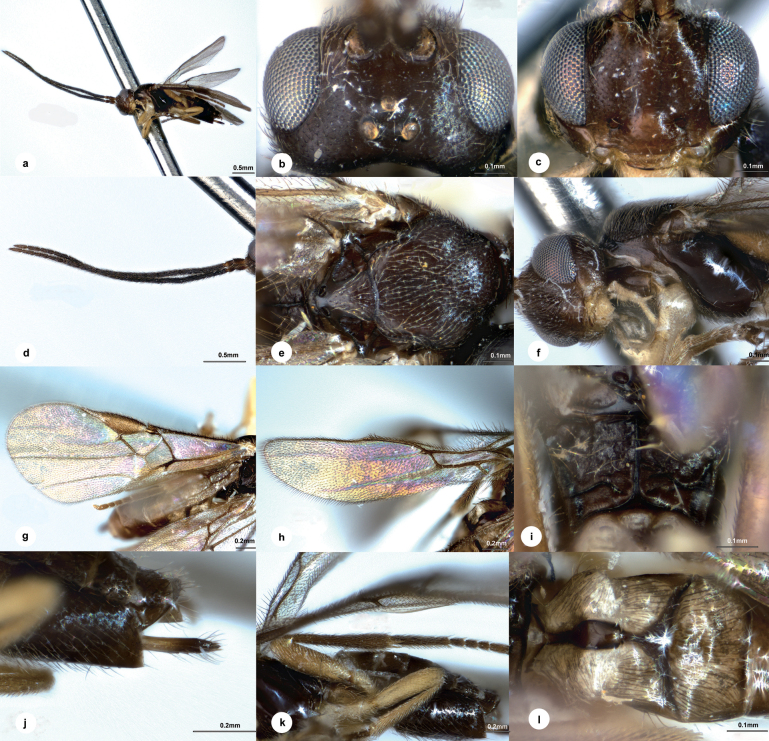
*Centistidealinearis* Liu & Polaszek, sp. nov., female, holotype **a** habitus, lateral view **b** head, dorsal view **c** head, frontal view **d** antenna **e** mesosoma, dorsal view **f** mesosoma, lateral view **g** fore wing **h** hind wing **i** propodeum **j** ovipositor sheath **k** hind leg **l** T1–T3, dorsal view.

**Female.** Body length 2.6 mm, fore wing length 2.6 mm (Fig. 3a).

***Head*.** Transverse in dorsal view, 1.7 × as wide as long, 1.5 × wider than mesoscutum. Eyes 2.0 × longer than temple in dorsal view (Fig. 3b). Temple a little shiny, with small setose punctures and with transverse wrinkles in between, not constricted behind eyes in dorsal view. Ocelli small, distance between fore and a hind ocellus 1.5 × longer than minor axis of a hind ocellus, POL:OD:OOL = 1.3:1.0:3.1. Frons flat and nearly polished except extremely fine transverse wrinkles. Vertex between eye and hind ocellus shiny and sculptured as temple. Face (Fig. 3c) shiny, with fine setose punctures, indistinctly convex medially, transverse, 1.3 × wider than high. Clypeus 1.6 × wider than medial length, weakly defined punctate. Length of malar space as long as basal width of mandible. Antenna (Fig. 3d) 1.2 × longer than body length, with scape, pedicel and 1^st^, 2^nd^, penultimate and ultimate flagellomeres 2.0, 1.7, 5.2, 4.9, 4.1 and 4.3 × longer than wide, 1^st^ about as long as 2^nd^, flagellomeres gradually shortened to apex.

***Mesosoma*.** Length:width:height = 10:4.2:6.3. Mesoscutum (Fig. 3e) shiny with superficial and extremely small punctures, intervals with extremely fine wrinkles, notauli distinct, crenulated near to anterior 1/2. Scutellar sulcus slightly curved, indistinctly crenulated. Scutellum strongly shiny, sculptured as mesoscutum, medio-posterior depressions large and oblong, widely separated, interval 3/4 of depression diameter). Propodeum (Fig. 3i) shiny, with distinct median carinae reaching beyond weakly defined costulae, anterior parts with indistinct punctures anteriorly except wrinkles elsewhere as posterior parts, anterior part 2.8 × longer than median length of metanotum. Mesopleuron (Fig. 3f) highly polished, impunctate.

***Legs*.** Hind femur (Fig. 3k) 3.3 × as long as its widest part. Length of hind femur:tibia:basitarsus = 1.5:1.9:1.0. Basitarsus of hind leg 0.9 × as long as tarsomeres 2–5.

***Wings*.** Fore wing (Fig. 3g): pterostigma, 2.6 × as long as its widest part; vein 1-R1 0.3 length of pterostigma; vein r:2-SR:2-M = 1.0:5.5:2.3, 1-SR:1-M = 1.0:4.1, 1-CU1:2-CU1 = 1.0:1.7; first discal cell of fore wing nearly 1.2 × wider than high. Hind wing (Fig. 3h): vein M+CU:1-M:r-m = 2.0:2.2:1.0.

***Metasoma*.** Indistinctly longer than mesosoma. T1 (Fig. 3l) highly polished, spatula-shaped, 2.8 × longer than its maximum width, distinctly narrowed anterior-medially. T2 transverse, 2.6 × wider than median length. T3 1.7 × longer than T2, weakly longitudinally striate. Hypopygium shorter than length of metasoma. Ovipositor sheath (Fig. 3j) 0.8 length of hind basitarsus, with long and dense setae apically.

***Colour*.** Dark brown, except metasoma more or less brown dorsally (Fig. 3a). Palpi and spurs pale yellow. Antenna and apical ovipositor sheath dark brown. Legs yellow to yellow-brown on all tarsi and hind tibia. Wing membrane hyaline, pterostigma brown, vein r, 2-SR, 1-SR, 1-M and 1-CU1 darker brown, other veins brown.

**Male.** Unknown.

##### Host.

Unknown.

##### Material examined

**(NHMUK). *Holotype***: • 1♀, Cameroon, Nkoémvon, D. Jackson, VII–VIII.1979, No. NHMUK010639762.

##### Distribution.

Cameroon.

##### Etymology.

The specific name “*linearis*” derives from the Latin, referring to the fine wrinkles on head and mesosoma.

##### Remarks.

This species is similar to *C.africana* but differs in the following: antenna 1.3 × longer than body (antenna shorter than body in *C.africana*); medio-posterior depressions on scutellum oblong, far away from each other, interval 3/4 of depression diameter (oval and close to each other in *C.africana*); and scutellar sulcus indistinctly crenulated (not crenulated or concave in *C.africana*).

#### ﻿ Centistidea longipedes

Taxon classificationAnimaliaHymenopteraBraconidae

Liu & Polaszek sp. nov.

A3DEFBBE-ACB2-56C1-BFD9-A1E2D859AA0F

https://zoobank.org/3164ADDF-C71B-4EB8-BB2A-A719EE211873

Fig. 4

##### Diagnosis.

Body length 2.2 mm, dark brown; head 1.7 × as wide as long, 1.3 × wider than mesoscutum; eyes 1.5 × longer than temple in dorsal view; temple with transverse wrinkles in between, not constricted behind eyes in dorsal view; distance between fore and a hind ocellus 1.2 × longer than minor axis of a hind ocellus, POL:OD:OOL = 1.2:1.0:3.2; frons flat and nearly polished except extremely fine transverse wrinkles; vertex between eye and hind ocellus shiny and sculptured as temple; face shiny, with extremely fine setose punctures, indistinctly convex medially, transverse, 1.4 × wider than high; clypeus 2.2 × wider than medial length, nearly polished; antenna 1.2 × longer than body length, with 1^st^, 2^nd^, penultimate and ultimate flagellomeres 6.3, 6.7, 3.8, and 4.6 × longer than wide, 1^st^ 1.1 × longer than 2^nd^; mesoscutum with superficial and extremely small punctures, intervals with extremely fine wrinkles, notauli obvious, crenulated to anterior 1/3; scutellar sulcus straight and crenulated; medio-posterior depressions on scutellum large and oblong, virtually touching each other; propodeum with distinct median carinae just reach costulae, anterior parts with indistinct punctures and several short rugae alongside median carinae, 2.5 × longer than median length of metanotum, posterior parts polished; hind leg extremely long, 2.6 × than metasoma; pterostigma narrow, 3.7 × as long as its widest part; vein 1-R1 0.3 of length of pterostigma; T1 poorly defined, 2.8 × longer than its maximum width; T2 1.2 × wider than median length; T3 0.9 × length of T2, not longitudinally striate.

##### Description.

**Figure 4.**
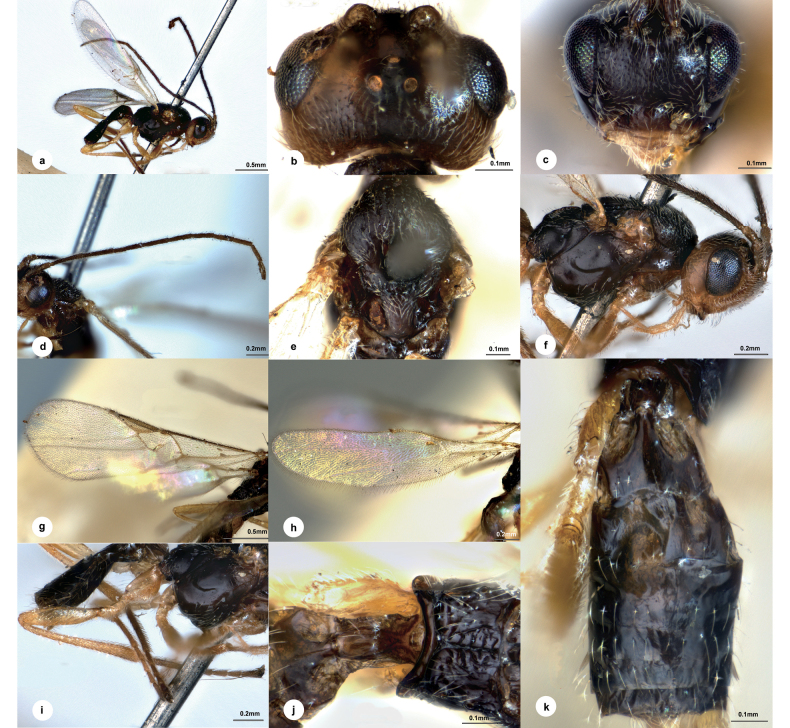
*Centistidealongipedes* Liu & Polaszek, sp. nov., male, holotype **a** habitus, lateral view **b** head, dorsal view **c** head, frontal view **d** antenna **e** mesosoma, dorsal view **f** mesosoma, lateral view **g** fore wing **h** hind wing **i** hind leg **j** propodeum and T1 **k** metasoma, dorsal view.

**Male.** Body length 2.2 mm, fore wing length 2.6 mm (Fig. 4a).

***Head*.** Transverse in dorsal view, 1.7 × as wide as long, 1.3 × wider than mesoscutum. Eyes 1.5 × longer than temple in dorsal view (Fig. 4b). Temple slightly shiny, small setose punctures with transverse wrinkles in between, not constricted behind eyes in dorsal view. Ocelli small, distance between fore and a hind ocellus 1.2 × longer than minor axis of a hind ocellus, POL:OD:OOL = 1.2:1.0:3.2. Frons flat and nearly polished except extremely fine transverse wrinkles. Vertex between eye and hind ocellus shiny and sculptured as temple. Face (Fig. 4c) shiny, with extremely fine setose punctures, indistinctly convex medially, transverse, 1.4 × wider than high. Clypeus 2.2 × wider than medial length, nearly polished. Length of malar space 1.6 × basal width of mandible. Antenna (Fig. 4d) 1.2 × longer than body length, with scape, pedicel and 1^st^, 2^nd^, penultimate and ultimate flagellomeres 2.3, 1.9, 6.3, 6.7, 3.8 and 4.6 × longer than wide, 1^st^ 1.1 × longer than 2^nd^, flagellomeres gradually shortened to apex.

***Mesosoma*.** Length:width:height = 10:4.6:6.5. Mesoscutum (Fig. 4e) shiny with superficial and extremely small punctures, intervals with extremely fine wrinkles, notauli obvious, crenulated to anterior 1/3. Scutellar sulcus slightly curved, crenulated. Scutellum shiny, sculptured as mesoscutum, medio-posterior depressions large and oblong, virtually touching each other. Propodeum (Fig. 4j) highly shiny, with distinct median carinae just reaching costulae, anterior parts with indistinct punctures and several short rugae alongside median carinae, 2.5 × longer than median length of metanotum, posterior parts polished. Mesopleuron (Fig. 4f) highly polished, impunctate.

***Legs*.** Hind leg (Fig. 4i) remarkedly long, 2.6 × than metasoma. Hind femur 4.1 × as long as its widest part. Length of hind femur:tibia:basitarsus = 2.2:3.2:1.0. Basitarsus of hind leg 0.6 × as long as tarsomeres 2–5.

***Wings*.** Fore wing (Fig. 4g): pterostigma narrow, 3.7 × as long as its widest part; vein 1-R1 0.3 of length of pterostigma; vein r:2-SR:2-M = 1.0:5.3:3.4, 1-SR:1-M = 1.0:4.0, 1-CU1:2-CU1 = 1.0:2.2; first discal cell of fore wing nearly 1/5 wider than high. Hind wing (Fig. 4h): vein M+CU:1M:1r-m = 1.5:1.9:1.0.

***Metasoma*.** Indistinctly longer than mesosoma. T1 (Fig. 4k) highly polished, poorly defined, spatula-shaped, 2.8 × longer than its maximum width, distinctly narrowed anterior-medially. T2 1.2 × wider than median length; T3 0.9 × length of T2, smooth without longitudinal striae.

***Colour*.** Dark brown (Fig. 4a). Palpi and spurs honey yellow. Antenna dark brown. Legs yellow, except apical 1/3 of hind tibia and hind tarsus brown. Wing membrane hyaline, pterostigma yellow-brown, vein r, 2-SR, 1-SR, 1-M and 1-CU1 yellow-brown, other veins pale.

**Female.** Unknown.

##### Host.

Unknown.

##### Material examined

**(NHMUK). *Holotype***: • 1♂, Cameroon, Mt Cameroon, Mann’s Quelle (2256 m), M Steele, 4.II.1932, B.M.1934-240, No. NHMUK010639754. ***Paratype***: • 1♂, Uganda, Ruwenzori Range, Bigo (3475 m), DS Fletcher, 20–22.VII.1952, No. NHMUK010639740.

##### Distribution.

Cameroon, Uganda.

##### Etymology.

The specific name “*longipedes*” derives from Latin, referring to the extremely long hind legs.

##### Remarks.

This species is similar to *C.leucopterae* (Wilkinson, 1936) but differs in the following: temple not constricted behind eyes in dorsal view (distinctly constricted in *C.leucopterae*); T2 1.2 × wider than median length (3.3 × wider in *C.leucopterae*); and T3 polished (longitudinally striate in *C.leucopterae*).

#### ﻿ Centistidea mubilibana

Taxon classificationAnimaliaHymenopteraBraconidae

(de Saeger, 1944)

91B869EC-9271-5B88-AD8A-2106785E5F5E

Fig. 5

Mirax mubilibana de Saeger, 1944: 34. Holotype in RMCA, examined.
Mirax mubilibana : Shenefelt 1973: 678.

##### Diagnosis.

Body length 2–3 mm, colour variable, mostly black; face, vertex and occiput very finely punctate, shiny; temple smooth; eyes a third longer than wide; length of the ocellar triangle approximately equal to the distance which separates it from each eye; antenna nearly as long as body, 1^st^ flagellomere a little longer and thinner than the following; mesoscutum regularly and finely punctate, more densely than the face, more sparsely laterally and disc of the scutellum, notauli present anteriorly; scutellar sulcus weakly arched, narrow and foveated; medio-posterior depressions of scutellum very small, round; propodeum rough, with carinae arranged as in *M.leucopterae*, but median carinae more wide, more or less divided, the apical area comprises approximately a third the length of propodeum; vein r almost completely absent; T1 2.5 × longer than its greatest width, striate or rugose; T2 smooth and shiny, with a small tubercle basal medially; T2 and T3 of the same length; T3 with slightly stronger longitudinal aciculation than T2; ovipositor sheath a little shorter than metatarsus III.

**Figure 5.**
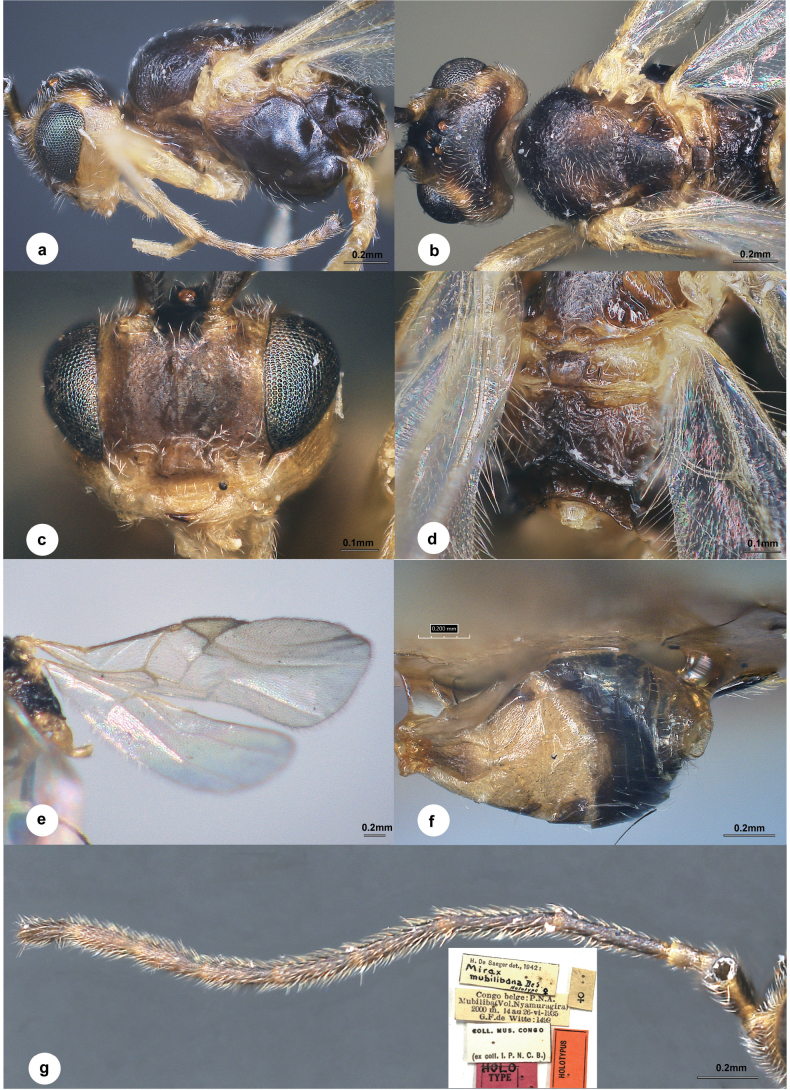
*Centistideamubilibana* (de Saeger, 1944), female, holotype **a** head and mesosoma, lateral view **b** head and mesosoma, dorsal view **c** face, frontal view **d** propodeum **e** wings **f** metasoma, dorsal view **g** antenna.

##### Host.

Unknown.

##### Material examined

**(RMCA). *Holotype***: • 1♀, Congo Belge: PNA Mubiliba (Vol. Nyamuragira), 2000 m, 14–26.VI.1935, G.F.de Witte: 1499, Coll. Mus. Congo (ex coll. I.P.N.C.B).

##### Distribution.

Democratic Republic of Congo, Rwanda.

#### ﻿ Centistidea tihamica

Taxon classificationAnimaliaHymenopteraBraconidae

Ahmad & Pandey, 2019

70253193-AB99-55E5-B097-57F0F96E16C0

Centistidea tihamica Ahmad & Pandey, 2019: 43. Holotype in the Insect Collection of the Department of Zoology, Aligarh Muslim University, Aligarh, India (not examined).

##### Diagnosis.

Body length 1.8 mm, mostly yellow-brown; length of eye 1.5 × temple in dorsal view; head and vertex indistinctly punctate; 1^st^ flagellomere 1.25 × longer than 2^nd^; penultimate flagellomere 2.5–3.0 × as long as wide; mesoscutum shiny with few distinct punctures, notauli only anteriorly impressed; scutellar sulcus distinct, present as a narrow groove and crenulated; medio-posterior depressions of scutellum semicircular and separated (from the original image); propodeum almost smooth with a complete median longitudinal carina bifurcate posteriorly, median carina of propodeum absent behind level of costulae; pterostigma with a long slender, apical expansion, 2.2 × longer than wide; vein r very prominent and 0.2 × as long as the height of pterostigma; vein 1-SR absent (from the original image); T1 4.0 × as long as its maximum width; T2 subtriangular, smooth, laterally membranous, and longitudinally striated; T3 longitudinally striated; ovipositor sheaths 0.15 × as long as fore wing (following Ghramh et al. 2019).

##### Host.

Unknown.

##### Distribution.

Saudi Arabia. Although not strictly in the Afrotropical region, the species is included here for future reference, in case it should eventually be discovered in the region.

##### Note.

No specimens were available for this study. Ghramh et al. (2019) described it as the first species of *Centistidea* from the Afrotropical region. However, when we examined the original descriptions and images and related specimens, all species originally described as *Mirax* including *africana*, *leucopterae*, and *mubilibana* in this area are all *Centistidea* by possessing medio-longitudinal carina on propodeum and more or less impressed notauli on anterior mesoscutum.

#### ﻿ Centistidea turneri

Taxon classificationAnimaliaHymenopteraBraconidae

Liu & Polaszek sp. nov.

46EEB38D-7199-5106-A1D0-2B31D7CB0DD1

https://zoobank.org/C0CE598F-E087-488B-AB29-87091DC0C8AD

Fig. 6

##### Diagnosis.

Body length 1.7 mm, light red-brown; eyes 1.8 × longer than temple in dorsal view; temple smooth, superficially punctate, a little constricted behind eyes in dorsal view; hind ocelli in a shallow depression, distance between fore and a hind ocellus 1.3 × longer than minor axis of a hind ocellus, POL:OD:OOL = 1.5:1.0:2.5; vertex between eye and hind ocellus shiny and polished; face polished, 1.4 × wider than high; antenna slightly shorter than body length, with penultimate and ultimate flagellomeres 2.2 and 2.5 × longer than wide, 1^st^ slightly longer (1.1 ×) than 2^nd^; mesoscutum with superficial and fine dense punctures anteriorly and laterally, more shallow and sparser dorsally, notauli hardly visible, only slightly depressed at anterior extremity; scutellar sulcus slightly curved, shallowly concave without crenulation; medio-posterior depressions of scutellum oblong, touching each other; propodeum with median carina reaching half way to hind margin, and bifurcated to two-thirds of lateral margin, largely polished elsewhere; pterostigma 2.8 × as long as its widest part, vein 1-R1 virtually absent; T1 polished, 2.5 × longer than its maximum width, strongly narrowed anterior-medially; T2 1.9 × wider than median length, not longitudinally striate at lateral membranous area; T3 1.4 × longer than T2, weakly longitudinally striate.

##### Description.

**Figure 6.**
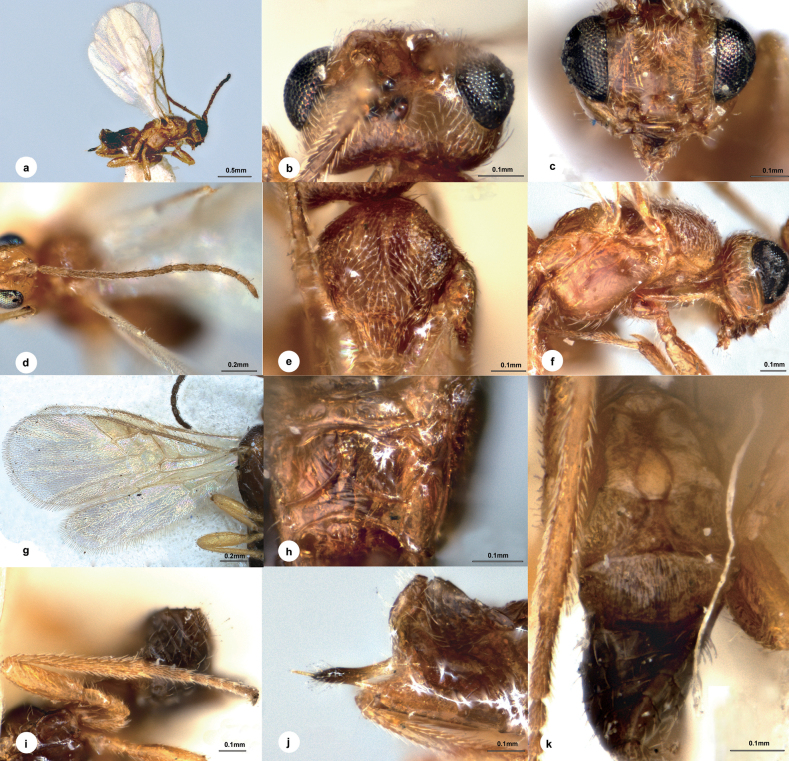
*Centistideaturneri* Liu & Polaszek, sp. nov., female, holotype **a** habitus, lateral view **b** head, dorsal view **c** head, frontal view **d** antenna **e** mesosoma, dorsal view **f** mesosoma, lateral view **g** wings **h** propodeum **i** hind leg **j** ovipositor sheath **k** metasoma, dorsal view.

**Female.** Body length 1.7 mm, fore wing length 1.8 mm (Fig. 6a).

***Head*.** Transverse in dorsal view, 1.8 × as wide as long, 1.3 × wider than mesoscutum. Eyes 1.8 × longer than temple in dorsal view (Fig. 6b). Temple slightly shiny, smooth, indistinctly punctate, sparsely pubescent, a little constricted behind eyes in dorsal view (Fig. 6c). Ocelli small, hind ocelli in a shallow depression, distance between fore and a hind ocellus 1.3 × longer than minor axis of a hind ocellus, POL:OD:OOL = 1.5:1.0:2.5. Frons flat and polished. Vertex between eye and hind ocellus shiny and polished. Face (Fig. 6c) indistinctly convex medially, transverse, 1.4 × wider than high. Clypeus 2.0 × wider than medial length, slightly polished. Length of malar space 1.5 × longer than width of mandible. Antenna (Fig. 6d) slightly shorter than body length, with scape, pedicel and 1^st^, 2^nd^, penultimate and ultimate flagellomeres 2.0, 1.6, 4.5, 4.5, 2.2, and 2.5 × longer than wide, 1^st^ slightly longer (1.1 ×) than 2^nd^, flagellomeres gradually shortened to penultimate flagellomeres.

***Mesosoma*.** Length:width:height = 1.8:1.0:1.3. Mesoscutum (Fig. 6e) shiny with superficial and fine dense punctures anteriorly and laterally, more shallow and sparser dorsally, notauli hardly visible dorsally, only slightly depressed at anterior extremity. Scutellar sulcus slightly curved, shallowly concave with crenulation. Scutellum shiny, sculptured as dorsal mesoscutum, medio-posterior depressions oblong, touching each other. Propodeum (Fig. 6h) shiny, with median carina reaching halfway to hind margin, and bifurcated to two thirds of lateral margin, largely polished elsewhere. Mesopleuron highly polished, impunctate.

***Legs*.** Hind femur (Fig. 6i) 3.3 × as long as its widest part. Length of hind femur:tibia:basitarsus = 1.9:2.9:1.0. Basitarsus of hind leg 0.7 × as long as tarsomeres 2–5.

***Wings*.** Fore wing (Fig. 6g): pterostigma 2.8 × as long as its widest part; vein 1-R1 virtually absent; vein r:2-SR:2-M = 1.0:6.7:3.0, 1-SR:1-M = 1.0:4.4, 1-CU1:2-CU1 = 1.0:1.6; first discal cell of fore wing indistinctly wider than high. Hind wing (Fig. 6g): vein M+CU:1-M:r-m = 2.0:2.5:1.0.

***Metasoma*.** Nearly as long as mesosoma. T1 (Fig. 6k) polished, spatula-shaped, 2.5 × longer than its maximum width, strongly narrowed antero-medially, transversely striate at lateral membranous area. T2 1.9 × wider than median length, not longitudinally striate at lateral membranous area. T3 1.4 × longer than T2, weakly longitudinally striate. Hypopygium not shorter than length of metasoma. Ovipositor sheath (Fig. 6j) 1.3 × longer than hind basitarsus, with long and dense setae at apical third.

***Colour*.** Light red-brown, terga posterior to T3 dark brown (Fig. 6a). Palpi and spurs light red-yellow. Antenna and basal half ovipositor sheath (apical half dark brow) yellow-brown. Legs yellow-brown except apical tarsomeres. Wing membrane hyaline, pterostigma pale yellow, vein 1-SR, 1-M and 1-CU1 brown, other veins pale yellow.

***Variation*.** Body colour varying from light yellow to dark brown on trunk of body among specimens. Specimens from Mossel Bay and Ceres from Cape Province tend to be darker when compared with the light yellow specimens from Pondoland of Port St. John in South Africa and Uganda. Body length varies from very small (1.2 mm) to large (2.2 mm).

**Male.** Similar to female, but body smaller with darker metasoma and longer antenna.

##### Host.

Leaf-miner in castor (*Ricinuscommunis*).

##### Material examined

**(NHMUK). *Holotype***: • 1♀, **SOUTH AFRICA**, Cape Province, Swellendam, RE Turner, II.1932, Brit. Mus 1932-145, No. NHMUK010639682. ***Paratypes***: • 1♀, South Africa, Cape Province, Somerset East, RE Turner, 23–31.XII.1930, Brit. Mus 1931-61, No. NHMUK010639720; • 2♀♀, South Africa, Cape Province, Mossel Bay, RE Turner, IV.1921, Brit. Mus 1921-210, Nos. NHMUK010639718, 010639716; • 1♀, same data except V.1921, Brit. Mus 1921-248, No. NHMUK010639719; • 6♀♀, same data except VI.1921, Brit. Mus 1921-294, Nos. NHMUK010639717, 010639706, 010639700, 010639710, 010639701, 010639686; • 2♀♀, same data except 5–31.VII.1921, Brit. Mus 1921-315, Nos. NHMUK010639715, 010639714; • 7♀♀, same data except VIII.1921, Brit. Mus 1921-353, Nos. NHMUK010639711, 010639712, 010639707, 010639708, 010639692, 010639687, 010639713; • 6♀♀, same data except IX.1921, Brit. Mus 1921-412, Nos. NHMUK010639705, 010639703, 010639695, 010639693, 010639697, 010639699; • 7♀♀1♂, same data except X.1921, Brit. Mus 1921-450, Nos. NHMUK010639696, 010639709, 010639683, 010639689, 010639694, 010639684, 010639691, 010639690; • 1♀, same data except 18–30.XI.1921, Brit. Mus 1922-2, No. NHMUK010639688; • 1♀, same data except I.1922, Brit. Mus 1922-67, No. NHMUK010639704; • 1♀, same data except II.1922, Brit. Mus 1922-97, No. NHMUK010639698; • 2♀♀, South Africa, Cape Province, Ceres (457 m), RE Turner, I.1921, Brit. Mus 1921-78, Nos. NHMUK010639685, 010639702; • 2♀♀, same data except II.1921, Brit. Mus 1921-115, Nos. NHMUK010639629 010639640; • 11♀♀, same data except III.1921, Brit. Mus 1921-150, Nos. NHMUK010639661, 010639654, 010639666, 010639650, 010639645, 010639674, 010639663, 010639655, 010639660, 010639671, 010639630; • 1♀, same data except II.1925, Brit. Mus 1925-116, No. NHMUK010639633; • 9♀♀, same data except III.1925, Brit. Mus 1925-161, Nos. NHMUK010639644, 010639638, 010639659, 010639636, 010639635, 010639651, 010639658, 010639668; • 1♀, same data except IV.1925, Brit. Mus 1925-210, No. NHMUK010639538; • 1♂, South Africa, Cape Province, Katberg (1219 m), RE Turner, X.1932, Brit. Mus 1932-521, No. NHMUK010639599; • 1♀, South Africa, Port St. John, Pondoland, RE Turner, 12–30.VI.1923, Brit. Mus 1923-363, No. NHMUK010639652; • 1♀, same data except 15–31.VIII.1923, Brit. Mus 1923-463, No. NHMUK010639541; • 1♀, same data except 1–13.III.1924, Brit. Mus 1924-177, No. NHMUK010639617; • 1♀1♂, same data except XII.1923, Brit. Mus 1924-54, Nos. NHMUK010639542, 010639549; • 1♀, same data except I.1924, Brit. Mus 1924-97, No. NHMUK010639605; • 1♀, same data except 29.I–5.II.1924, Brit. Mus 1924-109, No. NHMUK010639580; • 1♂, same data except 6-25.II.1924, Brit. Mus 1924-136, No. NHMUK010639557; • 1♀, same data except 18–31.III.1924, Brit. Mus 1924-191, No. NHMUK010639607; • 8♀♀, **Uganda**, Kampala, 7.V.1934, ex leaf-miner in castor (*Ricinuscommunis*), Nos. NHMUK010639764, 010639759, 010639742, 010639729, 010639749, 010639758, 010639755, 010639733.

##### Distribution.

South Africa, Uganda.

##### Etymology.

The specific name “*turneri*” expresses our gratitude to the late R.E. Turner for the large quantity of this species collected in South Africa.

##### Remarks.

From a short apical extension of the pterostigma to distinctly longer and equaling the length of pterostigma, vein 1-R1 is often present in *Centistidea*. In this species, however, it is absent compare to its Afrotropical allies; it is similar to C. *mubilibana* (de Saeger, 1944) for the carination of propodeum but differs in the following: length of the ocellar triangle nearly half of the distance which separates it from each eye (approximately equal in *C.mubilibana*); vein 1-SR present (almost completely absent in *C.mubilibana*); and T1 polished (striate or rugose in *C.mubilibana*).

## Supplementary Material

XML Treatment for Centistidea africana

XML Treatment for Centistidea areolaris

XML Treatment for Centistidea leucopterae

XML Treatment for Centistidea linearis

XML Treatment for Centistidea longipedes

XML Treatment for Centistidea mubilibana

XML Treatment for Centistidea tihamica

XML Treatment for Centistidea turneri

## ﻿Additional information

### Author ORCIDs

Zhen Liu https://orcid.org/0000-0002-8670-0205

Andrew Polaszek https://orcid.org/0000-0002-7171-3353

### Data availability

All of the data that support the findings of this study are available in the main.
